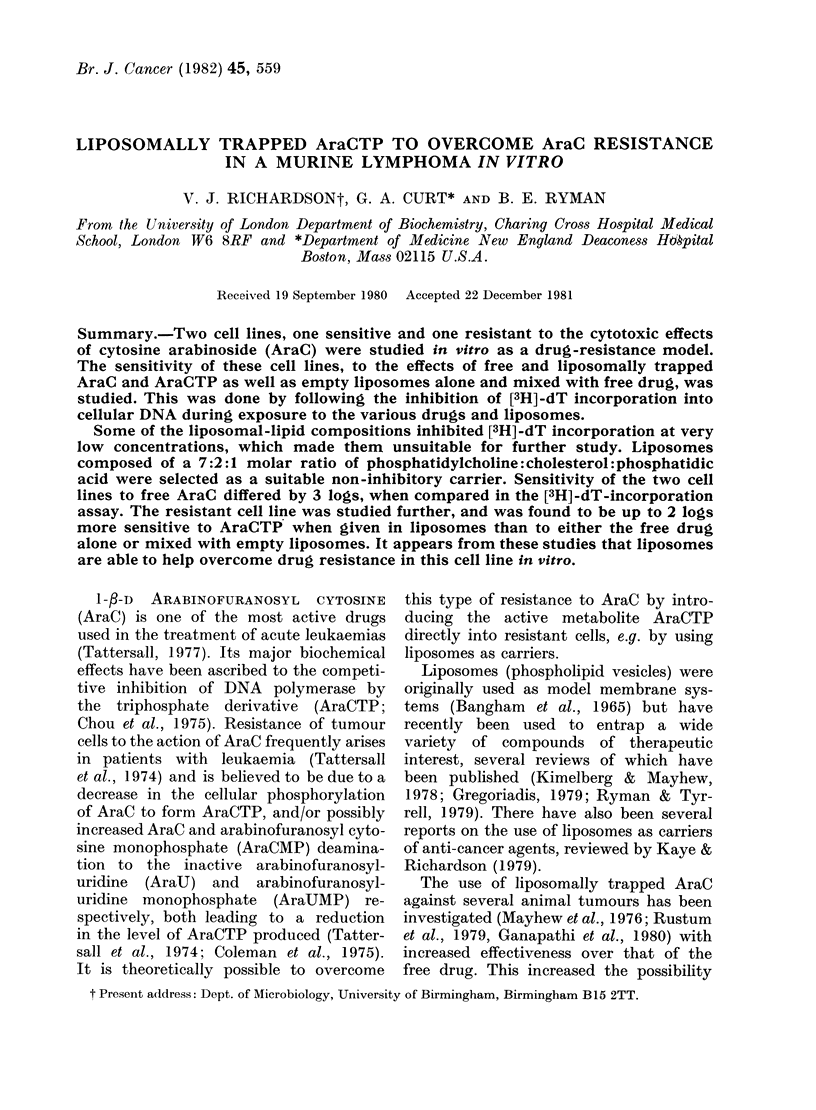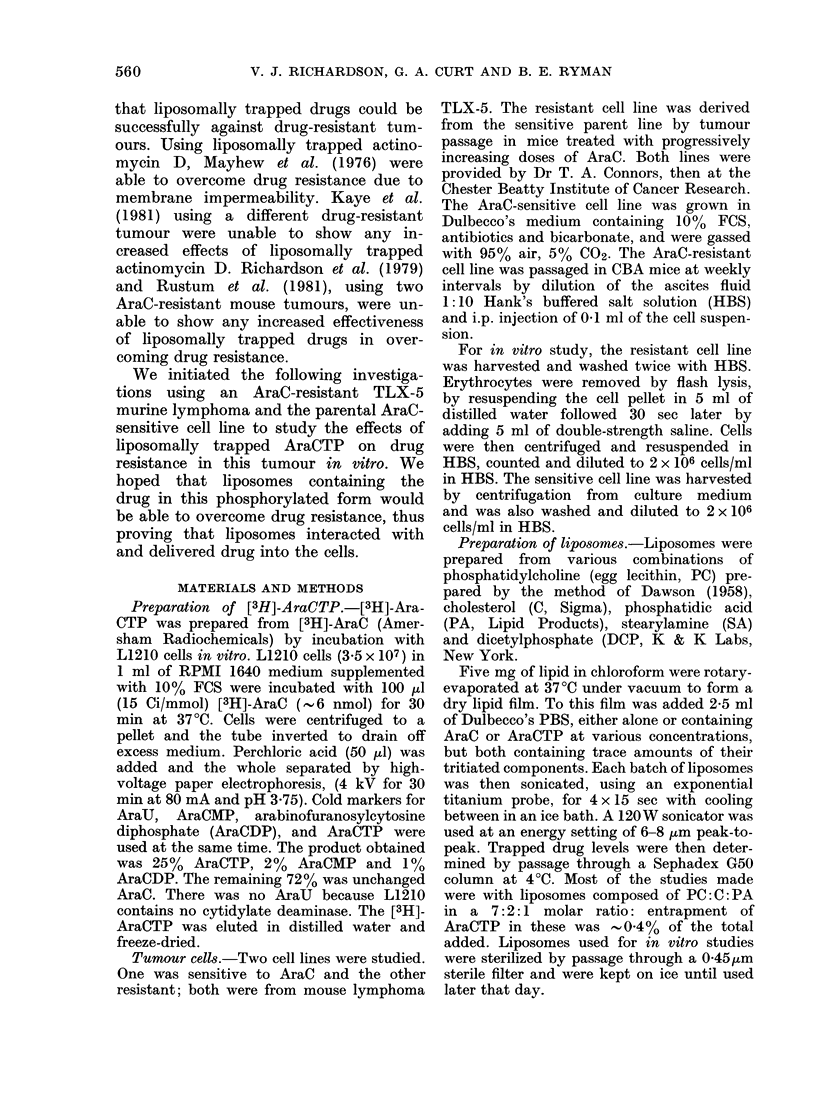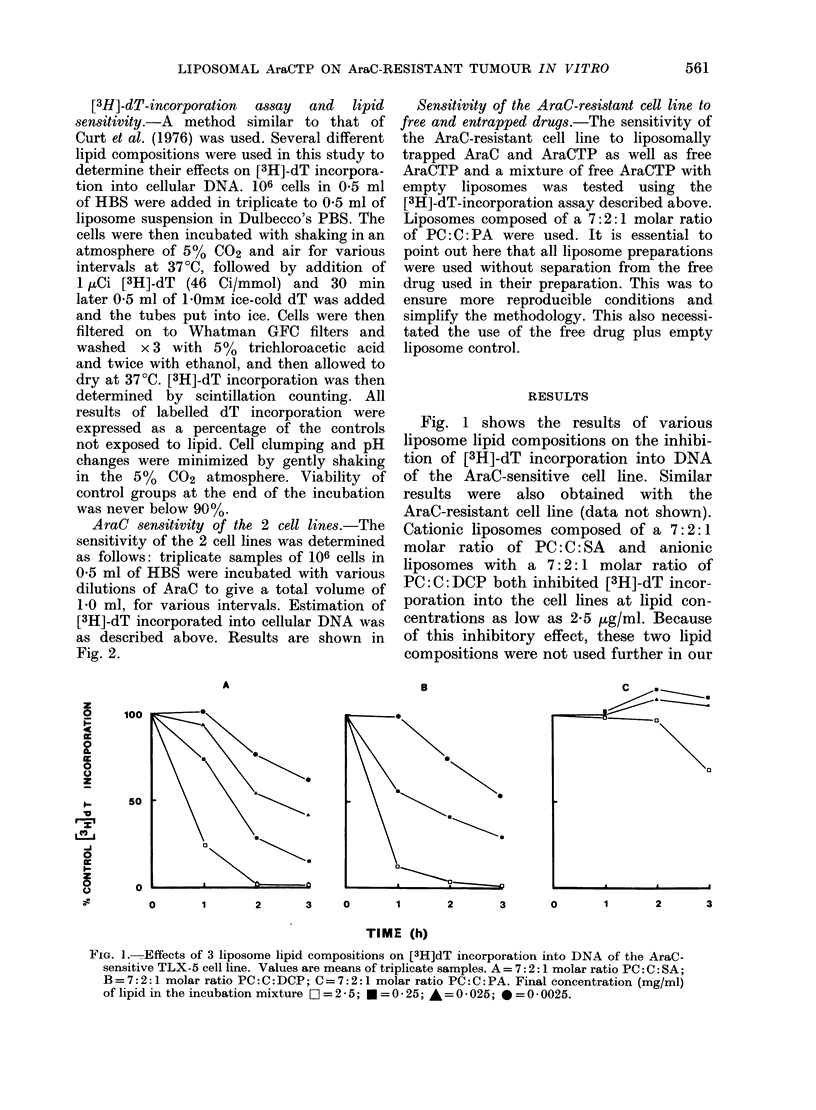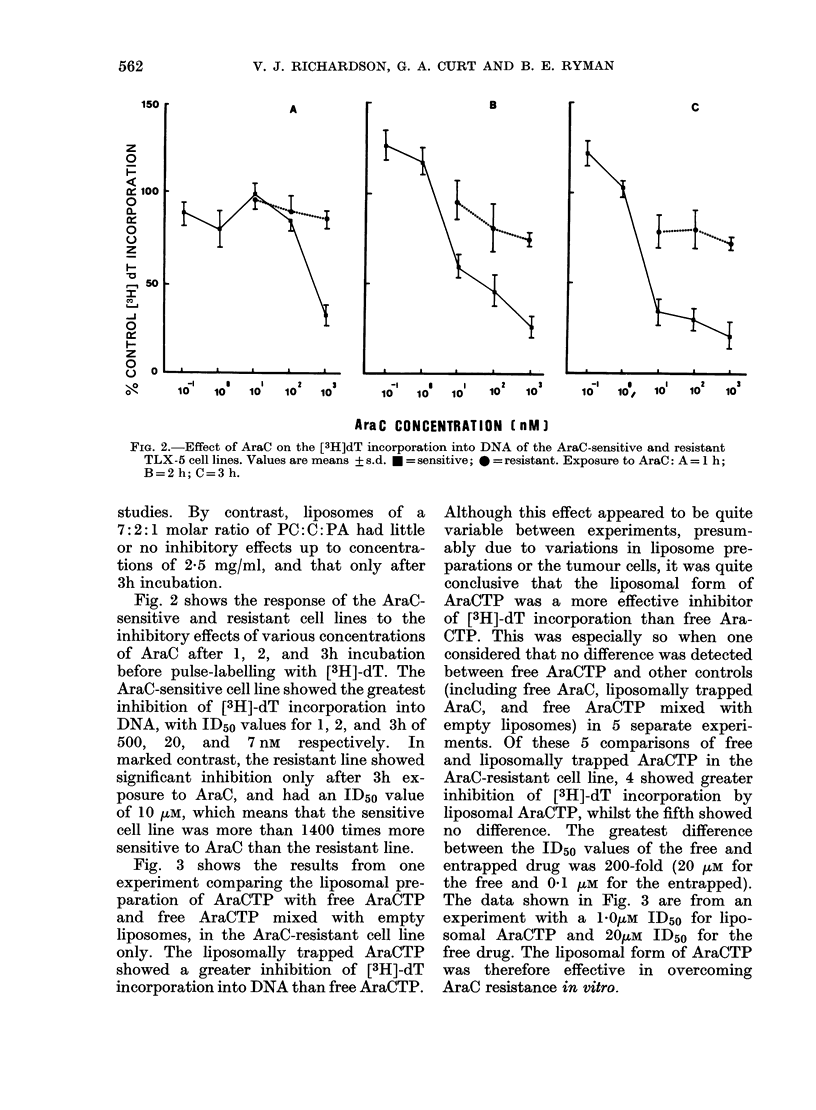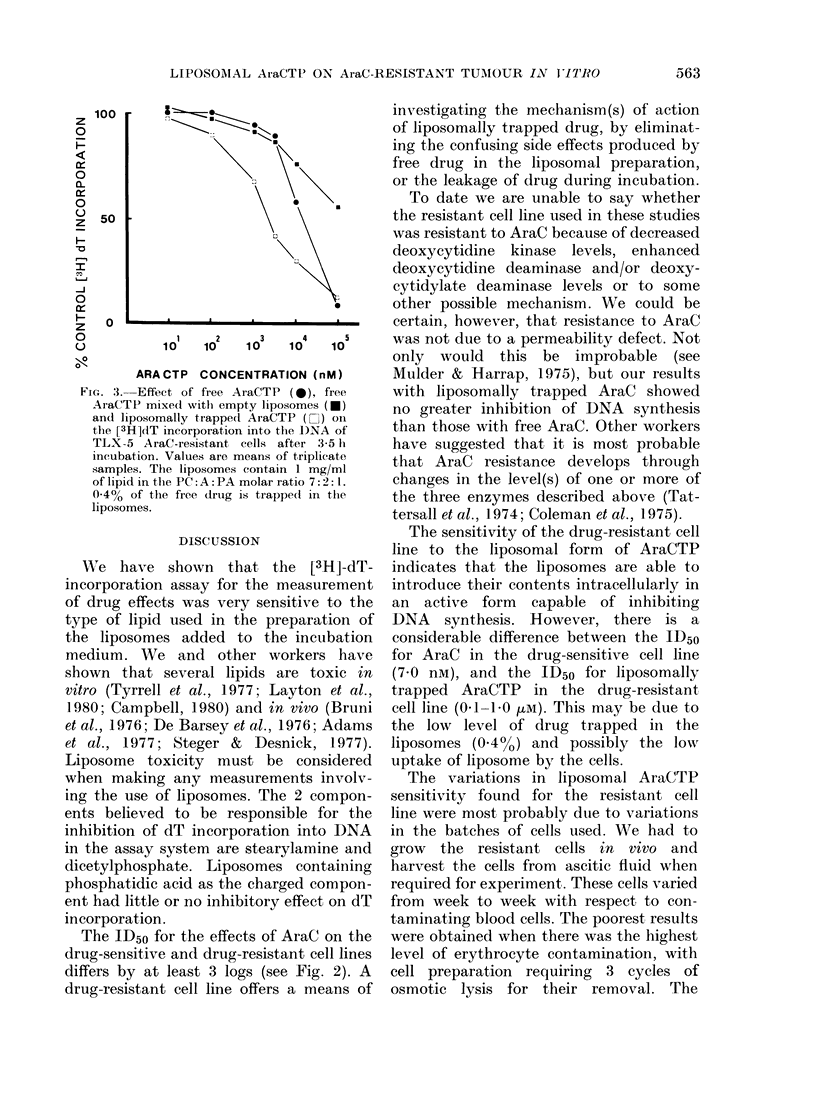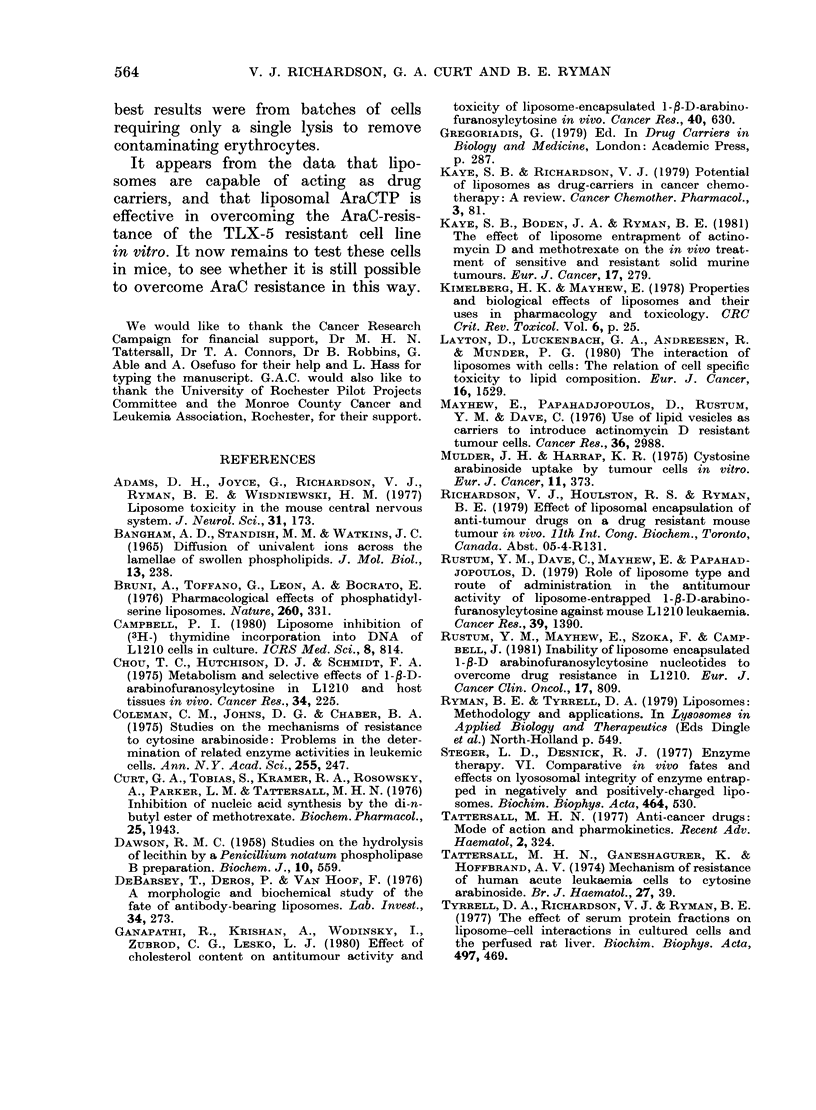# Liposomally trapped AraCTP to overcome AraC resistance in a murine lymphoma in vitro.

**DOI:** 10.1038/bjc.1982.92

**Published:** 1982-04

**Authors:** V. J. Richardson, G. A. Curt, B. E. Ryman

## Abstract

Two cell lines, one sensitive and one resistant to the cytotoxic effects of cytosine arabinoside (AraC) were studied in vitro as a drug-resistance model. The sensitivity of these cell lines, to the effects of free and liposomally trapped AraC and AraCTP as well as empty liposomes alone and mixed with free drug, was studied. This was done by following the inhibition of [3H]-dT incorporation into cellular DNA during exposure to the various drugs and liposomes. Some of the liposomal-lipid compositions inhibited [3H]-dT incorporation at very low concentrations, which made them unsuitable for further study. Liposomes composed of a 7:2:1 molar ratio of phosphatidylcholine:cholesterol:phosphatidic acid were selected as a suitable non-inhibitory carrier. Sensitivity of the two cell lines to free AraC differed by 3 logs, when compared in the [3H]-dT-incorporation assay. The resistant cell line was studied further, and was found to be up to 2 logs more sensitive to AraCTP when given in liposomes than to either the free drug alone or mixed with empty liposomes. It appears from these studies that liposomes are able to help overcome drug resistance in this cell line in vitro.


*Br. J. Cancer* (1982) **45**, 559

### LIPOSOMALLY TRAPPED AraCTP TO OVERCOME AraC RESISTANCE IN A MURINE LYMPHOMA *IN VITRO*

V. J. RICHARDSON†, G. A. CURT* AND B. E. RYMAN

*From the University of London Department of Biochemistry, Charing Cross Hospital Medical School, London W6 8RF and *Department of Medicine New England Deaconess Hospital Boston, Mass 02115 U.S.A.*

Received 19 September 1980 Accepted 22 December 1981

**Summary.—Two cell lines, one sensitive and one resistant to the cytotoxic effects of cytosine arabinoside (AraC) were studied *in vitro* as a drug-resistance model. The sensitivity of these cell lines, to the effects of free and liposomally trapped AraC and AraCTP as well as empty liposomes alone and mixed with free drug, was studied. This was done by following the inhibition of [$^3$H]-dT incorporation into cellular DNA during exposure to the various drugs and liposomes.**

**Some of the liposomal-lipid compositions inhibited [$^3$H]-dT incorporation at very low concentrations, which made them unsuitable for further study. Liposomes composed of a 7:2:1 molar ratio of phosphatidylcholine:cholesterol:phosphatidic acid were selected as a suitable non-inhibitory carrier. Sensitivity of the two cell lines to free AraC differed by 3 logs, when compared in the [$^3$H]-dT-incorporation assay. The resistant cell line was studied further, and was found to be up to 2 logs more sensitive to AraCTP when given in liposomes than to either the free drug alone or mixed with empty liposomes. It appears from these studies that liposomes are able to help overcome drug resistance in this cell line *in vitro*.**

1-β-D ARABINOFURANOSYL CYTOSINE (AraC) is one of the most active drugs used in the treatment of acute leukaemias (Tattersall, 1977). Its major biochemical effects have been ascribed to the competitive inhibition of DNA polymerase by the triphosphate derivative (AraCTP; Chou *et al.*, 1975). Resistance of tumour cells to the action of AraC frequently arises in patients with leukaemia (Tattersall *et al.*, 1974) and is believed to be due to a decrease in the cellular phosphorylation of AraC to form AraCTP, and/or possibly increased AraC and arabinofuranosyl cytosine monophosphate (AraCMP) deamination to the inactive arabinofuranosyl-uridine (AraU) and arabinofuranosyl-uridine monophosphate (AraUMP) respectively, both leading to a reduction in the level of AraCTP produced (Tattersall *et al.*, 1974; Coleman *et al.*, 1975). It is theoretically possible to overcome this type of resistance to AraC by introducing the active metabolite AraCTP directly into resistant cells, *e.g.* by using liposomes as carriers.

Liposomes (phospholipid vesicles) were originally used as model membrane systems (Bangham *et al.*, 1965) but have recently been used to entrap a wide variety of compounds of therapeutic interest, several reviews of which have been published (Kimelberg & Mayhew, 1978; Gregoriadis, 1979; Ryman & Tyrrell, 1979). There have also been several reports on the use of liposomes as carriers of anti-cancer agents, reviewed by Kaye & Richardson (1979).

The use of liposomally trapped AraC against several animal tumours has been investigated (Mayhew *et al.*, 1976; Rustum *et al.*, 1979, Ganapathi *et al.*, 1980) with increased effectiveness over that of the free drug. This increased the possibility

† Present address: Dept. of Microbiology, University of Birmingham, Birmingham B15 2TT.

that liposomally trapped drugs could be successfully against drug-resistant tumours. Using liposomally trapped actinomycin D, Mayhew *et al.* (1976) were able to overcome drug resistance due to membrane impermeability. Kaye *et al.* (1981) using a different drug-resistant tumour were unable to show any increased effects of liposomally trapped actinomycin D. Richardson *et al.* (1979) and Rustum *et al.* (1981), using two AraC-resistant mouse tumours, were unable to show any increased effectiveness of liposomally trapped drugs in overcoming drug resistance.

We initiated the following investigations using an AraC-resistant TLX-5 murine lymphoma and the parental AraC-sensitive cell line to study the effects of liposomally trapped AraCTP on drug resistance in this tumour *in vitro*. We hoped that liposomes containing the drug in this phosphorylated form would be able to overcome drug resistance, thus proving that liposomes interacted with and delivered drug into the cells.

### MATERIALS AND METHODS

*Preparation of [$^3$H]-AraCTP.*—[$^3$H]-AraCTP was prepared from [$^3$H]-AraC (Amersham Radiochemicals) by incubation with L1210 cells *in vitro*. L1210 cells ($3{\cdot}5 \times 10^7$) in 1 ml of RPMI 1640 medium supplemented with 10% FCS were incubated with 100 $\mu$l (15 Ci/mmol) [$^3$H]-AraC ($\sim$6 nmol) for 30 min at 37°C. Cells were centrifuged to a pellet and the tube inverted to drain off excess medium. Perchloric acid (50 $\mu$l) was added and the whole separated by high-voltage paper electrophoresis, (4 kV for 30 min at 80 mA and pH 3·75). Cold markers for AraU, AraCMP, arabinofuranosylcytosine diphosphate (AraCDP), and AraCTP were used at the same time. The product obtained was 25% AraCTP, 2% AraCMP and 1% AraCDP. The remaining 72% was unchanged AraC. There was no AraU because L1210 contains no cytidylate deaminase. The [$^3$H]-AraCTP was eluted in distilled water and freeze-dried.

*Tumour cells.*—Two cell lines were studied. One was sensitive to AraC and the other resistant; both were from mouse lymphoma TLX-5. The resistant cell line was derived from the sensitive parent line by tumour passage in mice treated with progressively increasing doses of AraC. Both lines were provided by Dr T. A. Connors, then at the Chester Beatty Institute of Cancer Research. The AraC-sensitive cell line was grown in Dulbecco's medium containing 10% FCS, antibiotics and bicarbonate, and were gassed with 95% air, 5% $CO_2$. The AraC-resistant cell line was passaged in CBA mice at weekly intervals by dilution of the ascites fluid 1:10 Hank's buffered salt solution (HBS) and i.p. injection of 0·1 ml of the cell suspension.

For *in vitro* study, the resistant cell line was harvested and washed twice with HBS. Erythrocytes were removed by flash lysis, by resuspending the cell pellet in 5 ml of distilled water followed 30 sec later by adding 5 ml of double-strength saline. Cells were then centrifuged and resuspended in HBS, counted and diluted to $2 \times 10^6$ cells/ml in HBS. The sensitive cell line was harvested by centrifugation from culture medium and was also washed and diluted to $2 \times 10^6$ cells/ml in HBS.

*Preparation of liposomes.*—Liposomes were prepared from various combinations of phosphatidylcholine (egg lecithin, PC) prepared by the method of Dawson (1958), cholesterol (C, Sigma), phosphatidic acid (PA, Lipid Products), stearylamine (SA) and dicetylphosphate (DCP, K & K Labs, New York.

Five mg of lipid in chloroform were rotary-evaporated at 37°C under vacuum to form a dry lipid film. To this film was added 2·5 ml of Dulbecco's PBS, either alone or containing AraC or AraCTP at various concentrations, but both containing trace amounts of their tritiated components. Each batch of liposomes was then sonicated, using an exponential titanium probe, for $4 \times 15$ sec with cooling between in an ice bath. A 120 W sonicator was used at an energy setting of 6–8 $\mu$m peak-to-peak. Trapped drug levels were then determined by passage through a Sephadex G50 column at 4°C. Most of the studies made were with liposomes composed of PC:C:PA in a 7:2:1 molar ratio: entrapment of AraCTP in these was $\sim$0·4% of the total added. Liposomes used for *in vitro* studies were sterilized by passage through a 0·45 $\mu$m sterile filter and were kept on ice until used later that day.

*[$^3$H]-dT-incorporation assay and lipid sensitivity.*—A method similar to that of Curt *et al.* (1976) was used. Several different lipid compositions were used in this study to determine their effects on [$^3$H]-dT incorporation into cellular DNA. $10^6$ cells in 0·5 ml of HBS were added in triplicate to 0·5 ml of liposome suspension in Dulbecco's PBS. The cells were then incubated with shaking in an atmosphere of 5% $CO_2$ and air for various intervals at 37°C, followed by addition of 1 $\mu$Ci [$^3$H]-dT (46 Ci/mmol) and 30 min later 0·5 ml of 1·0mM ice-cold dT was added and the tubes put into ice. Cells were then filtered on to Whatman GFC filters and washed ×3 with 5% trichloroacetic acid and twice with ethanol, and then allowed to dry at 37°C. [$^3$H]-dT incorporation was then determined by scintillation counting. All results of labelled dT incorporation were expressed as a percentage of the controls not exposed to lipid. Cell clumping and pH changes were minimized by gently shaking in the 5% $CO_2$ atmosphere. Viability of control groups at the end of the incubation was never below 90%.

*AraC sensitivity of the 2 cell lines.*—The sensitivity of the 2 cell lines was determined as follows: triplicate samples of $10^6$ cells in 0·5 ml of HBS were incubated with various dilutions of AraC to give a total volume of 1·0 ml, for various intervals. Estimation of [$^3$H]-dT incorporated into cellular DNA was as described above. Results are shown in Fig. 2.

*Sensitivity of the AraC-resistant cell line to free and entrapped drugs.*—The sensitivity of the AraC-resistant cell line to liposomally trapped AraC and AraCTP as well as free AraCTP and a mixture of free AraCTP with empty liposomes was tested using the [$^3$H]-dT-incorporation assay described above. Liposomes composed of a 7:2:1 molar ratio of PC:C:PA were used. It is essential to point out here that all liposome preparations were used without separation from the free drug used in their preparation. This was to ensure more reproducible conditions and simplify the methodology. This also necessitated the use of the free drug plus empty liposome control.

RESULTS

Fig. 1 shows the results of various liposome lipid compositions on the inhibition of [$^3$H]-dT incorporation into DNA of the AraC-sensitive cell line. Similar results were also obtained with the AraC-resistant cell line (data not shown). Cationic liposomes composed of a 7:2:1 molar ratio of PC:C:SA and anionic liposomes with a 7:2:1 molar ratio of PC:C:DCP both inhibited [$^3$H]-dT incorporation into the cell lines at lipid concentrations as low as 2·5 $\mu$g/ml. Because of this inhibitory effect, these two lipid compositions were not used further in our

FIG. 1.—Effects of 3 liposome lipid compositions on [$^3$H]dT incorporation into DNA of the AraC-sensitive TLX-5 cell line. Values are means of triplicate samples. A = 7:2:1 molar ratio PC:C:SA; B = 7:2:1 molar ratio PC:C:DCP; C = 7:2:1 molar ratio PC:C:PA. Final concentration (mg/ml) of lipid in the incubation mixture □ = 2·5; ■ = 0·25; ▲ = 0·025; ● = 0·0025.

FIG. 2.—Effect of AraC on the [$^3$H]dT incorporation into DNA of the AraC-sensitive and resistant TLX-5 cell lines. Values are means ± s.d. ■ = sensitive; ● = resistant. Exposure to AraC: A = 1 h; B = 2 h; C = 3 h.

studies. By contrast, liposomes of a 7:2:1 molar ratio of PC:C:PA had little or no inhibitory effects up to concentrations of 2·5 mg/ml, and that only after 3h incubation.

Fig. 2 shows the response of the AraC-sensitive and resistant cell lines to the inhibitory effects of various concentrations of AraC after 1, 2, and 3h incubation before pulse-labelling with [$^3$H]-dT. The AraC-sensitive cell line showed the greatest inhibition of [$^3$H]-dT incorporation into DNA, with $ID_{50}$ values for 1, 2, and 3h of 500, 20, and 7 nM respectively. In marked contrast, the resistant line showed significant inhibition only after 3h exposure to AraC, and had an $ID_{50}$ value of 10 $\mu$M, which means that the sensitive cell line was more than 1400 times more sensitive to AraC than the resistant line.

Fig. 3 shows the results from one experiment comparing the liposomal preparation of AraCTP with free AraCTP and free AraCTP mixed with empty liposomes, in the AraC-resistant cell line only. The liposomally trapped AraCTP showed a greater inhibition of [$^3$H]-dT incorporation into DNA than free AraCTP. Although this effect appeared to be quite variable between experiments, presumably due to variations in liposome preparations or the tumour cells, it was quite conclusive that the liposomal form of AraCTP was a more effective inhibitor of [$^3$H]-dT incorporation than free AraCTP. This was especially so when one considered that no difference was detected between free AraCTP and other controls (including free AraC, liposomally trapped AraC, and free AraCTP mixed with empty liposomes) in 5 separate experiments. Of these 5 comparisons of free and liposomally trapped AraCTP in the AraC-resistant cell line, 4 showed greater inhibition of [$^3$H]-dT incorporation by liposomal AraCTP, whilst the fifth showed no difference. The greatest difference between the $ID_{50}$ values of the free and entrapped drug was 200-fold (20 $\mu$M for the free and 0·1 $\mu$M for the entrapped). The data shown in Fig. 3 are from an experiment with a 1·0$\mu$M $ID_{50}$ for liposomal AraCTP and 20$\mu$M $ID_{50}$ for the free drug. The liposomal form of AraCTP was therefore effective in overcoming AraC resistance *in vitro*.

FIG. 3.—Effect of free AraCTP (●), free AraCTP mixed with empty liposomes (■) and liposomally trapped AraCTP (□) on the [$^3$H]dT incorporation into the DNA of TLX-5 AraC-resistant cells after 3·5 h incubation. Values are means of triplicate samples. The liposomes contain 1 mg/ml of lipid in the PC:A:PA molar ratio 7:2:1. 0·4% of the free drug is trapped in the liposomes.

### DISCUSSION

We have shown that the [$^3$H]-dT-incorporation assay for the measurement of drug effects was very sensitive to the type of lipid used in the preparation of the liposomes added to the incubation medium. We and other workers have shown that several lipids are toxic *in vitro* (Tyrrell *et al.*, 1977; Layton *et al.*, 1980; Campbell, 1980) and *in vivo* (Bruni *et al.*, 1976; De Barsey *et al.*, 1976; Adams *et al.*, 1977; Steger & Desnick, 1977). Liposome toxicity must be considered when making any measurements involving the use of liposomes. The 2 components believed to be responsible for the inhibition of dT incorporation into DNA in the assay system are stearylamine and dicetylphosphate. Liposomes containing phosphatidic acid as the charged component had little or no inhibitory effect on dT incorporation.

The $ID_{50}$ for the effects of AraC on the drug-sensitive and drug-resistant cell lines differs by at least 3 logs (see Fig. 2). A drug-resistant cell line offers a means of investigating the mechanism(s) of action of liposomally trapped drug, by eliminating the confusing side effects produced by free drug in the liposomal preparation, or the leakage of drug during incubation.

To date we are unable to say whether the resistant cell line used in these studies was resistant to AraC because of decreased deoxycytidine kinase levels, enhanced deoxycytidine deaminase and/or deoxycytidylate deaminase levels or to some other possible mechanism. We could be certain, however, that resistance to AraC was not due to a permeability defect. Not only would this be improbable (see Mulder & Harrap, 1975), but our results with liposomally trapped AraC showed no greater inhibition of DNA synthesis than those with free AraC. Other workers have suggested that it is most probable that AraC resistance develops through changes in the level(s) of one or more of the three enzymes described above (Tattersall *et al.*, 1974; Coleman *et al.*, 1975).

The sensitivity of the drug-resistant cell line to the liposomal form of AraCTP indicates that the liposomes are able to introduce their contents intracellularly in an active form capable of inhibiting DNA synthesis. However, there is a considerable difference between the $ID_{50}$ for AraC in the drug-sensitive cell line (7·0 nM), and the $ID_{50}$ for liposomally trapped AraCTP in the drug-resistant cell line (0·1–1·0 μM). This may be due to the low level of drug trapped in the liposomes (0·4%) and possibly the low uptake of liposome by the cells.

The variations in liposomal AraCTP sensitivity found for the resistant cell line were most probably due to variations in the batches of cells used. We had to grow the resistant cells *in vivo* and harvest the cells from ascitic fluid when required for experiment. These cells varied from week to week with respect to contaminating blood cells. The poorest results were obtained when there was the highest level of erythrocyte contamination, with cell preparation requiring 3 cycles of osmotic lysis for their removal. The best results were from batches of cells requiring only a single lysis to remove contaminating erythrocytes.

It appears from the data that liposomes are capable of acting as drug carriers, and that liposomal AraCTP is effective in overcoming the AraC-resistance of the TLX-5 resistant cell line *in vitro*. It now remains to test these cells in mice, to see whether it is still possible to overcome AraC resistance in this way.

We would like to thank the Cancer Research Campaign for financial support, Dr M. H. N. Tattersall, Dr T. A. Connors, Dr B. Robbins, G. Able and A. Osefuso for their help and L. Hass for typing the manuscript. G.A.C. would also like to thank the University of Rochester Pilot Projects Committee and the Monroe County Cancer and Leukemia Association, Rochester, for their support.

### REFERENCES

ADAMS, D. H., JOYCE, G., RICHARDSON, V. J., RYMAN, B. E. & WISDNIEWSKI, H. M. (1977) Liposome toxicity in the mouse central nervous system. *J. Neurol. Sci.*, **31**, 173.

BANGHAM, A. D., STANDISH, M. M. & WATKINS, J. C. (1965) Diffusion of univalent ions across the lamellae of swollen phospholipids. *J. Mol. Biol.*, **13**, 238.

BRUNI, A., TOFFANO, G., LEON, A. & BOCRATO, E. (1976) Pharmacological effects of phosphatidylserine liposomes. *Nature*, **260**, 331.

CAMPBELL, P. I. (1980) Liposome inhibition of ($^3$H-) thymidine incorporation into DNA of L1210 cells in culture. *ICRS Med. Sci.*, **8**, 814.

CHOU, T. C., HUTCHISON, D. J. & SCHMIDT, F. A. (1975) Metabolism and selective effects of 1-β-D-arabinofuranosylcytosine in L1210 and host tissues *in vivo*. *Cancer Res.*, **34**, 225.

COLEMAN, C. M., JOHNS, D. G. & CHABER, B. A. (1975) Studies on the mechanisms of resistance to cytosine arabinoside: Problems in the determination of related enzyme activities in leukemic cells. *Ann. N.Y. Acad. Sci.*, **255**, 247.

CURT, G. A., TOBIAS, S., KRAMER, R. A., ROSOWSKY, A., PARKER, L. M. & TATTERSALL, M. H. N. (1976) Inhibition of nucleic acid synthesis by the di-*n*-butyl ester of methotrexate. *Biochem. Pharmacol.*, **25**, 1943.

DAWSON, R. M. C. (1958) Studies on the hydrolysis of lecithin by a *Penicillium notatum* phospholipase B preparation. *Biochem. J.*, **10**, 559.

DEBARSEY, T., DEROS, P. & VAN HOOF, F. (1976) A morphologic and biochemical study of the fate of antibody-bearing liposomes. *Lab. Invest.*, **34**, 273.

GANAPATHI, R., KRISHAN, A., WODINSKY, I., ZUBROD, C. G., LESKO, L. J. (1980) Effect of cholesterol content on antitumour activity and toxicity of liposome-encapsulated 1-β-D-arabinofuranosylcytosine *in vivo*. *Cancer Res.*, **40**, 630.

GREGORIADIS, G. (1979) Ed. In *Drug Carriers in Biology and Medicine*, London: Academic Press, p. 287.

KAYE, S. B. & RICHARDSON, V. J. (1979) Potential of liposomes as drug-carriers in cancer chemotherapy: A review. *Cancer Chemother. Pharmacol.*, **3**, 81.

KAYE, S. B., BODEN, J. A. & RYMAN, B. E. (1981) The effect of liposome entrapment of actinomycin D and methotrexate on the *in vivo* treatment of sensitive and resistant solid murine tumours. *Eur. J. Cancer*, **17**, 279.

KIMELBERG, H. K. & MAYHEW, E. (1978) Properties and biological effects of liposomes and their uses in pharmacology and toxicology. *CRC Crit. Rev. Toxicol.* Vol. **6**, p. 25.

LAYTON, D., LUCKENBACH, G. A., ANDREESEN, R. & MUNDER, P. G. (1980) The interaction of liposomes with cells: The relation of cell specific toxicity to lipid composition. *Eur. J. Cancer*, **16**, 1529.

MAYHEW, E., PAPAHADJOPOULOS, D., RUSTUM, Y. M. & DAVE, C. (1976) Use of lipid vesicles as carriers to introduce actinomycin D resistant tumour cells. *Cancer Res.*, **36**, 2988.

MULDER, J. H. & HARRAP, K. R. (1975) Cystosine arabinoside uptake by tumour cells *in vitro*. *Eur. J. Cancer*, **11**, 373.

RICHARDSON, V. J., HOULSTON, R. S. & RYMAN, B. E. (1979) Effect of liposomal encapsulation of anti-tumour drugs on a drug resistant mouse tumour *in vivo*. *11th Int. Cong. Biochem., Toronto, Canada*. Abst. 05-4-R131.

RUSTUM, Y. M., DAVE, C., MAYHEW, E. & PAPAHADJOPOULOS, D. (1979) Role of liposome type and route of administration in the antitumour activity of liposome-entrapped 1-β-D-arabinofuranosylcytosine against mouse L1210 leukaemia. *Cancer Res.*, **39**, 1390.

RUSTUM, Y. M., MAYHEW, E., SZOKA, F. & CAMPBELL, J. (1981) Inability of liposome encapsulated 1-β-D arabinofuranosylcytosine nucleotides to overcome drug resistance in L1210. *Eur. J. Cancer Clin. Oncol.*, **17**, 809.

RYMAN, B. E. & TYRRELL, D. A. (1979) Liposomes: Methodology and applications. In *Lysosomes in Applied Biology and Therapeutics* (Eds Dingle *et al.*) North-Holland p. 549.

STEGER, L. D., DESNICK, R. J. (1977) Enzyme therapy. VI. Comparative *in vivo* fates and effects on lyososomal integrity of enzyme entrapped in negatively and positively-charged liposomes. *Biochim. Biophys. Acta*, **464**, 530.

TATTERSALL, M. H. N. (1977) Anti-cancer drugs: Mode of action and pharmokinetics. *Recent Adv. Haematol*, **2**, 324.

TATTERSALL, M. H. N., GANESHAGURER, K. & HOFFBRAND, A. V. (1974) Mechanism of resistance of human acute leukaemia cells to cytosine arabinoside. *Br. J. Haematol.*, **27**, 39.

TYRRELL, D. A., RICHARDSON, V. J. & RYMAN, B. E. (1977) The effect of serum protein fractions on liposome–cell interactions in cultured cells and the perfused rat liver. *Biochim. Biophys. Acta*, **497**, 469.